# Parallel fiber and climbing fiber responses in rat cerebellar cortical neurons *in vivo*

**DOI:** 10.3389/fnsys.2013.00016

**Published:** 2013-05-17

**Authors:** Dan-Anders Jirenhed, Fredrik Bengtsson, Henrik Jörntell

**Affiliations:** Department of Experimental Medical Science, Lund UniversityLund, Sweden

**Keywords:** cerebellum, Purkinje cells, Golgi cells, interneurons, long-term depression, long-term potentiation

## Abstract

Over the last few years we have seen a rapidly increasing interest in the functions of the inhibitory interneurons of the cerebellar cortex. However, we still have very limited knowledge about their physiological properties *in vivo*. The present study provides the first description of their spontaneous firing properties and their responses to synaptic inputs under non-anesthetized conditions in the decerebrated rat *in vivo*. We describe the spike responses of molecular layer interneurons (MLI) in the hemispheric crus1/crus2 region and compare them with those of Purkinje cells (PCs) and Golgi cells (GCs), both with respect to spontaneous activity and responses evoked by direct electrical stimulation of parallel fibers (PFs) and climbing fibers (CFs). In agreement with previous findings in the cat, we found that the CF responses in the interneurons consisted of relatively long lasting excitatory modulations of the spike firing. In contrast, activation of PFs induced rapid but short-lasting excitatory spike responses in all types of neurons. We also explored PF input plasticity in the short-term (10 min) using combinations of PF and CF stimulation. With regard to *in vivo* recordings from cerebellar cortical neurons in the rat, the data presented here provide the first demonstration that PF input to PC can be potentiated using PF burst stimulation and they suggest that PF burst stimulation combined with CF input may lead to potentiation of PF inputs in MLIs. We conclude that the basic responsive properties of the cerebellar cortical neurons in the rat *in vivo* are similar to those observed in the cat and also that it is likely that similar mechanisms of PF input plasticity apply.

## Introduction

The cerebellar cortical circuitry receives (excitatory) input from mossy fiber (MF) synapses on granule cells, which in turn send their output via ascending axons and subsequently the parallel fibers (PFs) into the molecular layer (Eccles et al., 1967; Ito, 1984). There the PFs make excitatory synaptic connections onto molecular layer interneurons (MLI) and the dendrites of both Purkinje cells (PCs) and Golgi cells (GCs) (Eccles et al., 1967; Ito, 1984). The MLI in turn make inhibitory connections onto PCs, which individually may be quite weak *in vivo* (Bengtsson et al., 2012), while the GCs make inhibitory synapses on granule cells (Eccles et al., 1967; Ito, 2006). In addition, the PCs each receive powerful excitatory input from one climbing fiber (CF) (Eccles et al., 1967; Ito, 1984). More recently, it was found that also MLIs receive CF input (Jorntell and Ekerot, 2003; Szapiro and Barbour, 2007) and possibly GCs (Sugihara et al., 1999, 2001), but see Galliano et al. (2013).

Crus I/crus II in the posterior lobe of the rat cerebellar cortex receives its CF input mainly from the rostral parts of the dorsal accessory and medial accessory inferior olive (rDAO and rMAO). The MF inputs are supplied from the basilar pontine nuclei (Serapide et al., 2001; Holtzman et al., 2009) and from the lateral reticular nucleus, LRN (Wu et al., 1999; Xu et al., 2012). The crus I/crus II region has been studied extensively by several research groups in recent years. These studies have provided much information about the general anatomical organization of MF and CF inputs, and the responses of granule cells, GCs, PCs, and MLIs (Chadderton et al., 2004; Pijpers et al., 2005, 2006; Holtzman et al., 2006a,b, 2009; Wise et al., 2010; Tahon et al., 2011; Chu et al., 2012; Duguid et al., 2012). Important data on the range of PC simple spike activity during behavior have been presented in two studies of this region (Bryant et al., 2010; Cao et al., 2012). In addition, some of the response properties of rodent cerebellar cortical cells *in vivo* have been characterized in a series of elegant studies in the mouse (Barmack and Yakhnitsa, 2008, 2011), although this study was carried out in the vestibulo-cerebellum. However, none of these studies characterized the specific responses of the MLIs to PF and CF inputs.

The PF input system converges with CF input on the PC dendritic tree and soma, where the CF input can regulate the polarity of plasticity at the PF-PC synapses, with paired PF and CF input causing long-term depression (Ito et al., 1982; Jorntell and Hansel, 2006) [see also Safo and Regehr (2008)]. Such CF induced PF plasticity in the PC has long been regarded the most important learning mechanism in the cerebellar cortex (Marr, 1969; Albus, 1971; Ito, 1984, 2006; Dean et al., 2010). However, there is also PF-CF convergence at MLIs and the PF-MLI synapses can undergo CF-dependent plasticity as well, but with the reverse polarities relative to the PF-PC synapses. These opposite plasticity effects are seen even though both PF-MLI and PF-PC synapses are excitatory synapses formed by the same type of axon (Jorntell and Ekerot, 2002, 2003; Rancillac and Crepel, 2004; Dean et al., 2010; Jorntell et al., 2010; Gao et al., 2012).

In non-anesthetized rodents *in vivo*, however, there has so far been no characterization of the general firing properties of MLIs, or MLI responses to direct PF and CF stimulation, respectively. Neither have plastic effects in MLIs and PCs of repeated combination of these inputs been explored. Therefore, in the present investigation we wish to characterize the spontaneous activities in cerebellar cortical cells and their responses to direct afferent stimulation, and also to explore whether a combination of these inputs results in plastic effects in the PF inputs to MLIs and PCs under non-anesthetized conditions.

## Results

### Properties of the spontaneous neuronal activity

**Figure 1:**
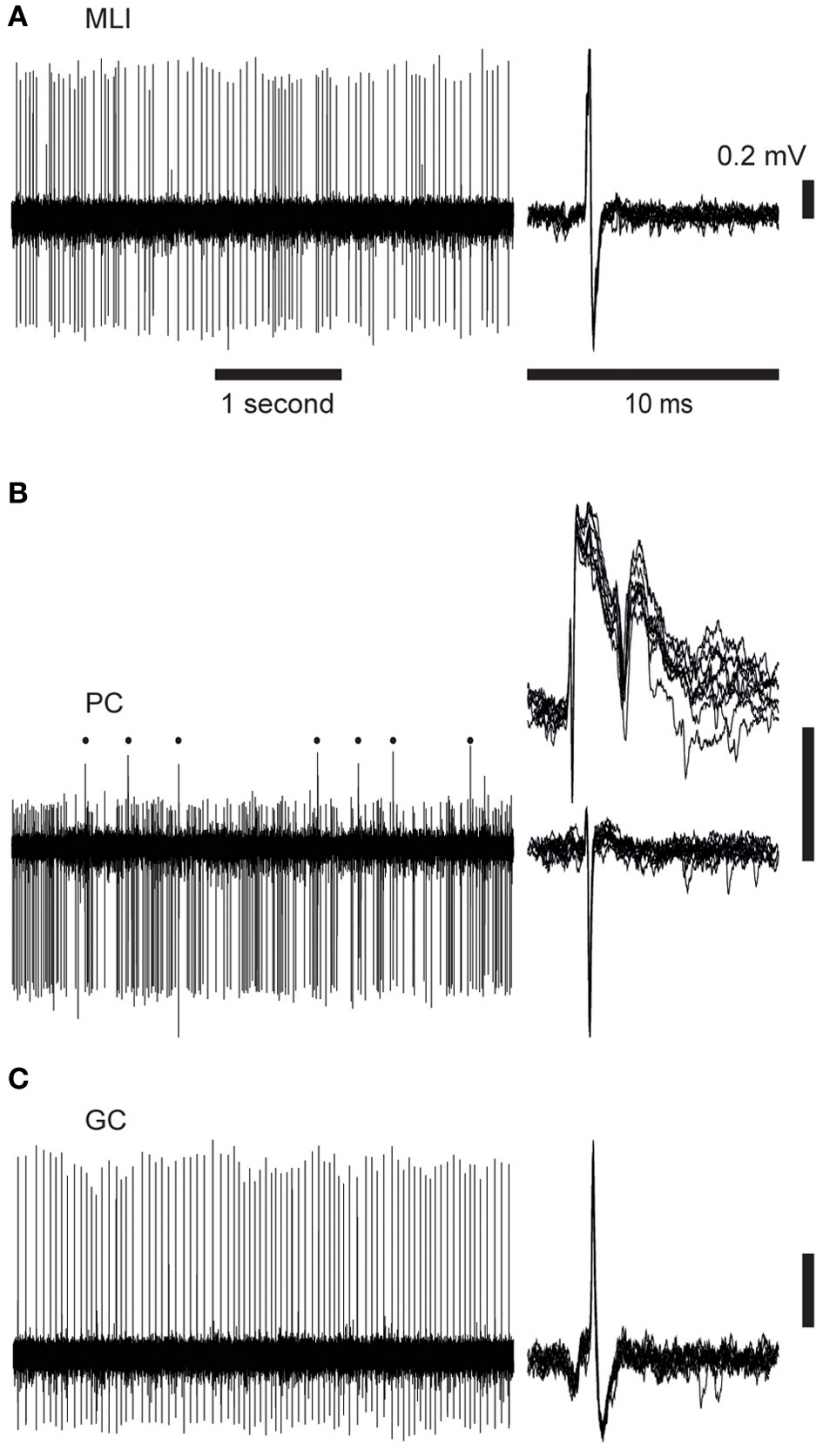
**Cell activity records.** Time scales are 1 s (left) and 10 ms (right). The panels to the left illustrate raw spontaneous activity, the right hand panels illustrate 10 superimposed spikes. Vertical scale bars indicate 0.2 mV potential. **(A)** Spontaneous spike activity of a molecular layer interneuron (MLI). **(B)** Purkinje cell (PC) with complex spikes (upper right panel and indicated by dots in the left panel) and simple spikes (lower right). **(C)** Spike activity of a Golgi cell (GC).

We made recordings in the superficial part of crus 2, 1 mm lateral to the paravermal vein, i.e., the same recording regions as in Bengtsson and Jorntell (2007), in which the CF responses to electrical stimulation of the snout correspond to responses of the type found in the C2 zone (Jorntell et al., 2000). When the metal microelectrode was advanced from the surface of the cerebellum, the characteristic background activities of the molecular layer, PC layer, and granule cell layer, respectively, were readily monitored and were combined with the electronic monitoring of the recording depth to verify the cortical layer recorded from. Spike recordings obtained from neurons located above the first PC layer, and which lacked complex spikes, were classified as MLI; see Figure 1A for illustrated example. Neurons recorded in the PC layer, and which exhibited complex spikes, were classified as PCs; illustrated in Figure 1B. For PCs, the present analysis concerns only the simple spike responses, with the exception of the complex spikes evoked from stimulation of the inferior olive (IO), as described below. Note that we did not observe a prominent simple spike pause after the complex spikes, in agreement with previous findings from the non-anesthetized decerebrated cat (Bengtsson et al., 2011) and awake monkeys (Miall et al., 1998). We also recorded activity from 6 large neurons (judging by the depth of more than 50 μm across which the spikes could be recorded) within the first 0.2 mm beneath the first PC layer (see Figure 1C for illustration). The latter neuron was tentatively classified as GCs, supported by additional quantitative analysis of their spike firing patterns (see below).

**Figure 2:**
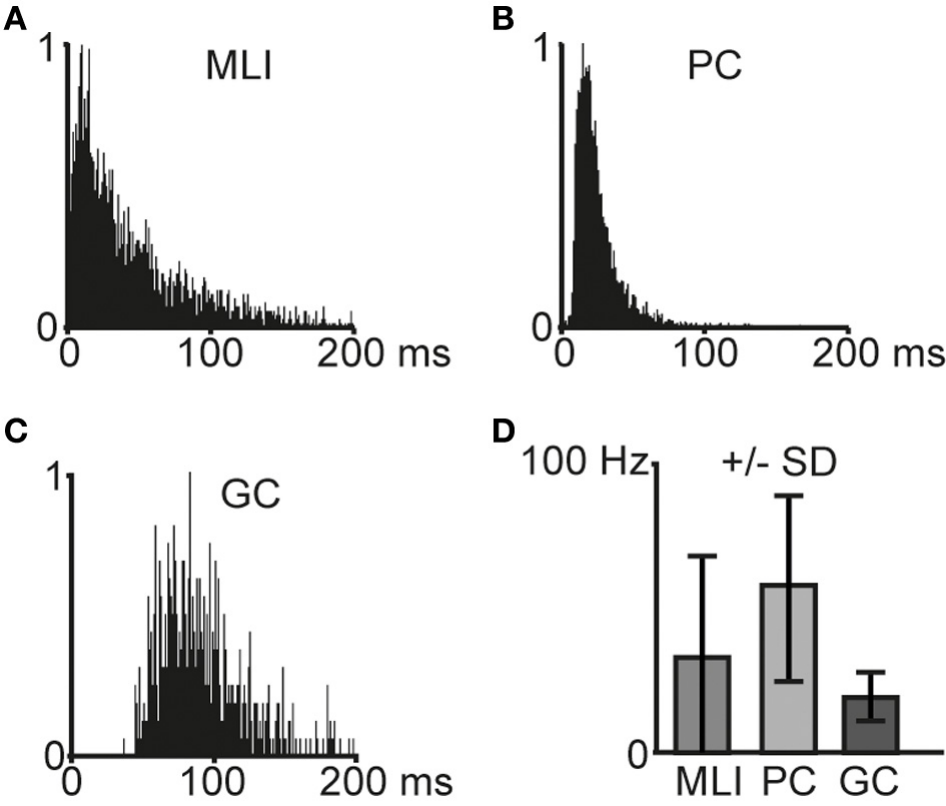
**Characteristics of spontaneous spike activity in cerebellar cortical neurons. (A**–**C)** Frequency distribution histograms that illustrate interspike intervals in example cells (*N* = 1 for each histogram). Bin width is 1 ms. **(A)** Molecular layer interneuron (MLI), **(B)** Purkinje cell (PC), and **(C)** Golgi cell (GC). **(D)** Average spontaneous spike frequencies for molecular layer interneurons (MLI, *N* = 11), Purkinje cells (PC, *N* = 6), and Golgi cells (GC, *N* = 5). Error bars indicate ± one standard deviation.

Interspike interval (ISI) histograms for the different cell types are illustrated in Figures 2A–C. The average spontaneous spike activity levels are illustrated in Figure 2D and specified in Table 1.

**Table 1:** **Descriptive statistical measures for different cell types**.

**Cell type**	**CVlog**	**Avg freq**	**CV2**	**ISIperc05**	**ISImed**
MLI (*N* = 11)	0.25 ± 0.20	32.9 ± 34.6	0.68 ± 0.22	0.015 ± 0.011	0.044 ± 0.033
PC (*N* = 8)	0.24 ± 0.04	56.4 ± 31.5	0.58 ± 0.09	0.006 ± 0.003	0.018 ± 0.010
GC (*N* = 6)	0.10 ± 0.07	19.1 ± 8.5	0.35 ± 0.18	0.035 ± 0.018	0.058 ± 0.027

For details of the selection and computation of analyzed parameters see Methods. All data are presented as average ± SD.

As the tentative GCs could potentially have been granule cells, Lugaro cells or Globular interneurons (Hirono et al., 2012), we further quantified the spike firing of GCs. The median spike interval of the GCs was 58 ± 27 ms, which is comparable to identified GCs (Holtzman et al., 2006b) recorded in the same region but using anesthetized rats, although overall our GCs had shorter median ISIs. Additional quantifications (CVlog = 0.10 ± 0.07; CV2 = 0.35 ± 0.18; ISI 5th percentile = 35 ± 18 ms) indicated that these cells were similar to GCs morphologically identified by Ruigrok et al. (2011), again using anesthetized rats but in vestibulocerebellum instead of crus 2. The statistics for all cell types are summarized in Table 1.

When comparing MLIs to GCs, the relative differences in all measures are comparable to the results presented by Ruigrok et al. (2011). PCs displayed the highest spontaneous spike frequency, followed by MLIs and the GCs. The most regular spiking, as measured by CVlog and CV2, was found in GCs, whereas MLIs were found to display the highest degree of irregularity in the spontaneous spiking activity. Overall, our cells had a somewhat higher level of spiking activity compared to Ruigrok et al. (2011).

### Properties of responses evoked by PF stimulation

**Figure 3:**
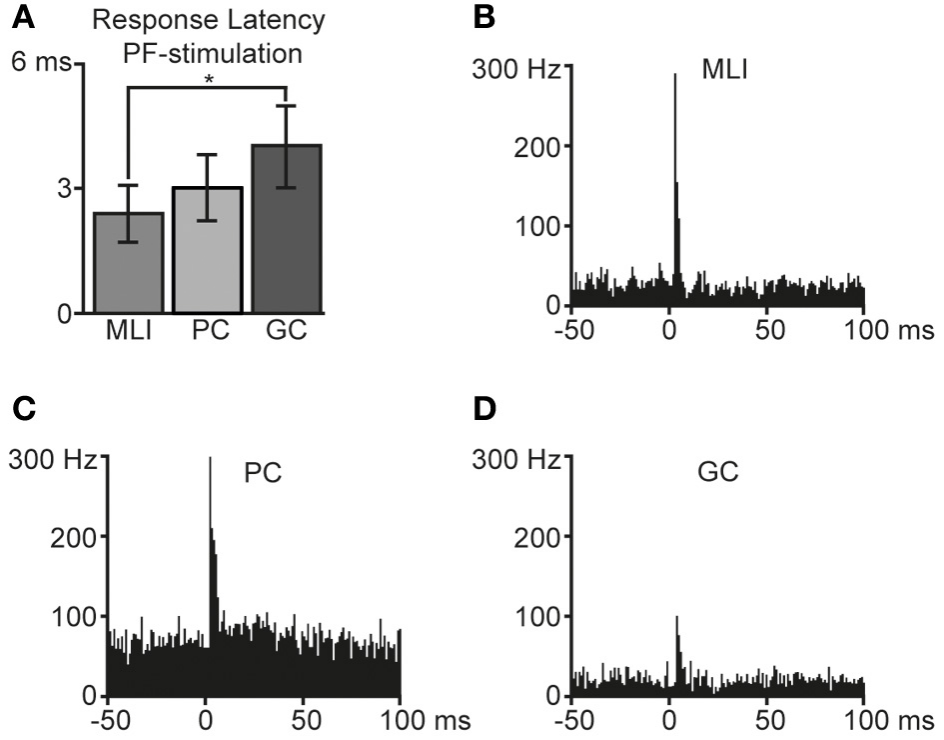
**Responses to parallel fiber (PF) stimulation.** Bin width is 1 ms. **(A)** Average response latencies for molecular layer interneurons (MLI, *N* = 11), Purkinje cell (PC, *N* = 6) simple spikes and Golgi cells (GC, *N* = 5). Error bars indicate ± one standard deviation. The difference in response latencies between MLIs and GCs was significant (two-tailed *t*-test, *p* < 0.05). Averaged peristimulus time histograms of responses to PF-stimulation are illustrated in **(B)** for molecular layer interneurons, **(C)** Purkinje cells (simple spikes), and in **(D)** Golgi cells.

Using tungsten-in-glass microelectrodes placed in the superficial molecular layer some 0.2 mm away from the recording electrode, we stimulated PFs and recorded the responses in the different types of neurons. All PF stimulations were made at intensities below 25 μA. In agreement with our previous description of powerful unitary EPSPs evoked by PF stimulation in MLIs in the cat *in vivo* (Jorntell and Ekerot, 2003), the MLIs in the present study responded with powerful excitatory responses to PF stimulation (illustrated in Figures 3A,B) with an average response latency of 2.4 ± 0.5 ms and a duration of 1.6 ± 0.7 ms (*N* = 11). PCs also responded strongly to the PF stimulation (Figure 3C) with an average response latency of 3.0 ± 0.8 ms and a duration of 2.0 ± 1.0 ms (*N* = 8). Compared to MLIs, the GCs (Figure 3D) had significantly longer (*p* < 0.05) response latencies of 4.0 ± 1.0 ms (*N* = 6), in agreement with their relatively slow and moderate PF EPSCs recorded *in vitro* (Dieudonne, 1998; Kanichay and Silver, 2008). Furthermore, GCs also displayed a trend (*p* = 0.055) to longer response durations (2.4 ± 0.5 ms) in comparison with MLIs.

### Properties of responses evoked by CF stimulation

**Figure 4:**
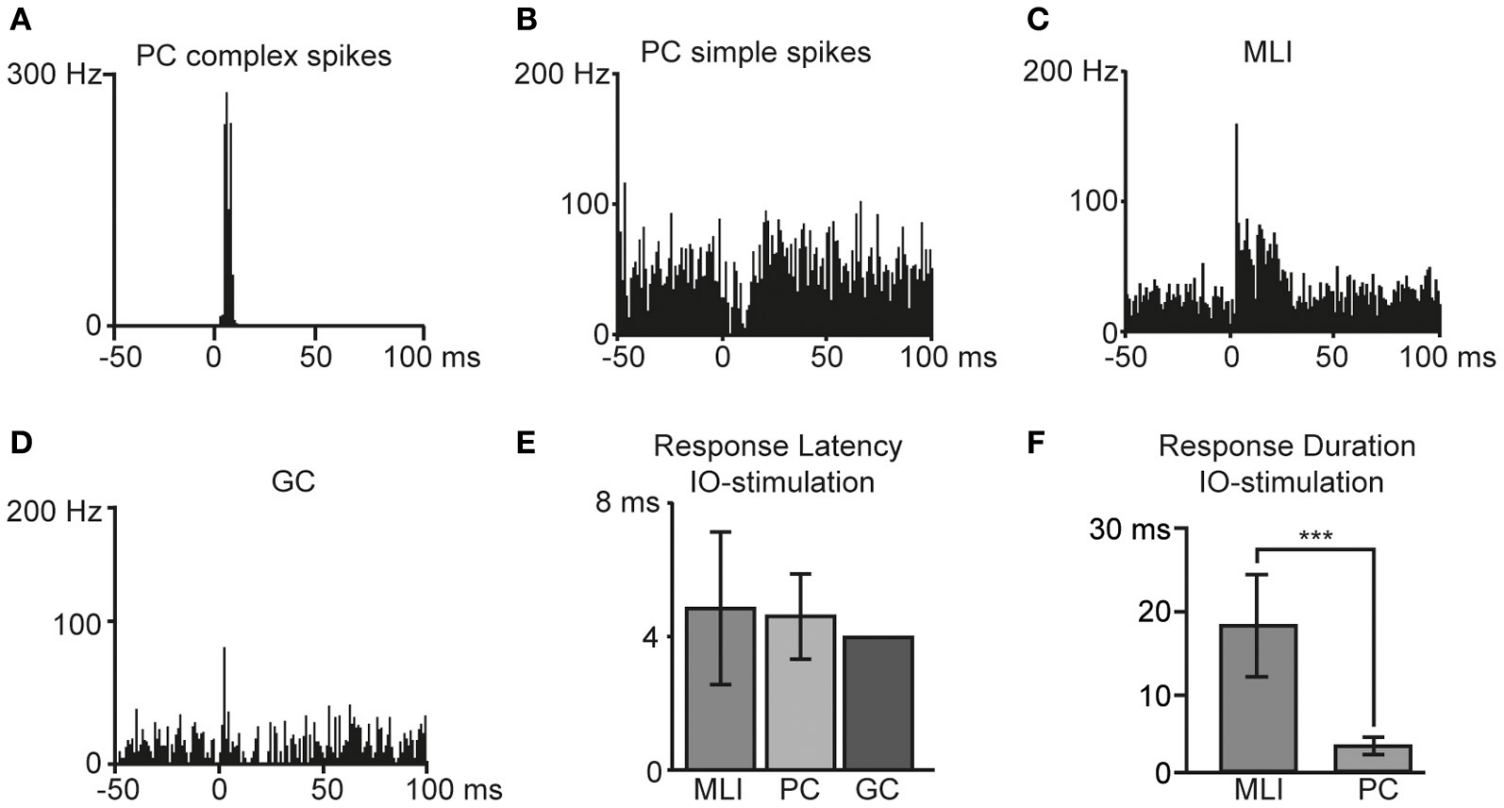
**Responses to inferior olive (IO) stimulation.** Bin width is 1 ms. Peristimulus time histograms of example cells in response to IO-stimulation are illustrated; in **(A)** Purkinje cell complex spikes, **(B)** Purkinje cell simple spikes, **(C)** molecular layer interneurons and in **(D)** a Golgi cell. **(E)** Average response latencies for molecular layer interneurons (MLI), Purkinje cell (PC) complex spikes, and one Golgi cell (GC). **(F)** Duration of IO evoked responses in MLIs and PCs. The difference was statistically significant (two-tailed *t*-test, *p* < 0.001). Error bars indicate ± one standard deviation.

Another tungsten-in-glass microelectrode placed in the IO was used to evoke CF activity. The stimulation electrode was verified to evoke CF field responses and PC complex spike responses in the recording region at stimulation intensities well below 100 μA (Figure 4A). With regard to the PC simple spikes, stimulation of the IO did not evoke any net excitation (Figure 4B), similar to the IO-evoked responses described in the decerebrated cat (Bengtsson et al., 2011; Jorntell and Ekerot, 2011). This is expected, since an appropriate IO stimulation should only activate CF input and no mossy fiber input to the PC. The slight inhibitory effect on the PC simple spike activity (Figure 4B) could be explained by CF activation of MLIs and/or the complex spike-evoked simple spike pause.

As previously reported in the cat *in vivo* (Jorntell and Ekerot, 2002, 2003) and in rat cerebellar slices (Szapiro and Barbour, 2007), also MLIs displayed distinct CF responses (Figure 4C). In agreement with observations in the cat (Jorntell and Ekerot, 2003), and in line with the long duration of putative CF responses of interneurons *in vitro* (Szapiro and Barbour, 2007), we found that CF activation evoked relatively long-lasting excitatory spike responses in rat MLIs (Figure 4C). The excitatory MLI response to IO stimulation had a mean duration of 17 ± 6 ms, defined as the time period where spike activity was greater than the spontaneous frequency by +1 SD. Responses to IO stimulation were also recorded from one GC (Figure 4D) that displayed a response latency of 4.0 ms. The response latencies observed in the different cell types are summarized in Figure 4E. The response duration in MLIs was significantly (*p* < 0.001) greater than that of the complex spike response in the PCs (Figure 4F).

### PF response changes after PF burst and PF + IO stimulation protocols

**Figure 5:**
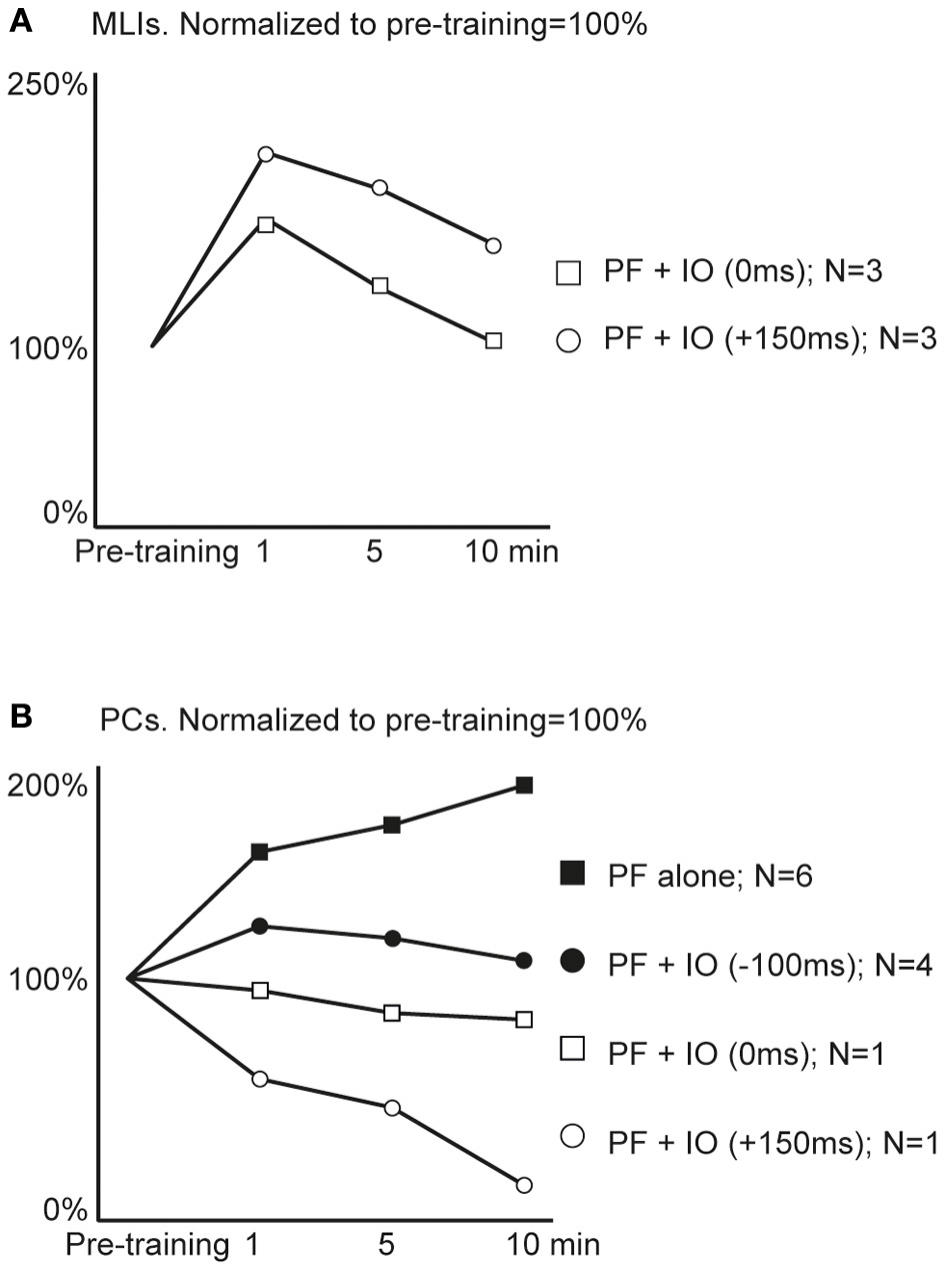
**Average effects of plasticity protocols.** Responses to parallel fiber (PF) stimulation were quantified before and after application of different one minute stimulation protocols. **(A)** Molecular layer interneuron responses before application of a stimulation protocol (Pre-training) and subsequent responses to single PF stimulus pulses at 1, 5 and 10 min after training with two different protocols. *N* denotes the number of cells observed for each stimulation protocol. **(B)** Purkinje cell simple spike responses as above, before and after training with four different protocols.

**Figure 6:**
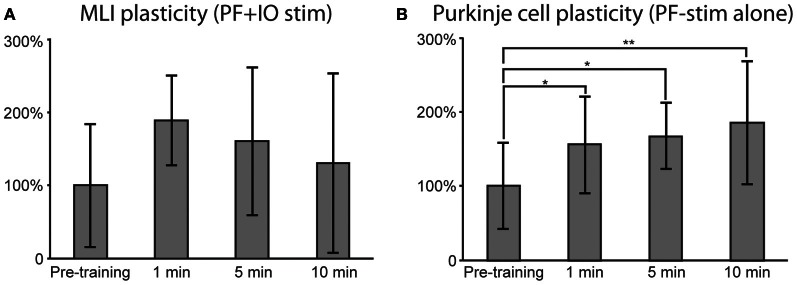
**Effects of plasticity protocols.** Similar to Figure 5, responses to PF stimulation were quantified before and after the stimulation protocols. **(A)** For MLIs, mean responses after pooling the data from both plasticity protocols (*N* = 6). A repeated measures ANOVA revealed a trend, though not a significant effect (*p* = 0.06). **(B)** PC responses after PF alone plasticity protocol (*N* = 6). A repeated measure ANOVA followed by *post-hoc* Tukey tests revealed statistically significant differences between pre-training and all post-training time points. In **(A)** and **(B)**, error bars indicate ± one standard deviation.

Using electrical PF stimulation *in vivo*, the PF input to PCs and MLIs has been shown to be bi-directionally modifiable or plastic (Jorntell and Ekerot, 2002, 2003; Ekerot and Jorntell, 2003) and similar findings have been made in the rat *in vitro* for PCs (Lev-Ram et al., 2002, 2003; Coesmans et al., 2004; Belmeguenai and Hansel, 2005) and for MLIs (Rancillac and Crepel, 2004). The classical demonstration of LTD in PCs used simultaneous activation of PF and CF inputs (Ito et al., 1982; Ekerot and Kano, 1985). But a later study has shown that the CF can be activated in a time window of approximately −250 to +250 ms relative to the onset of PF activation and still produce LTD (Safo and Regehr, 2008). Here, we wanted first to explore whether the basic plasticity effects of PF inputs are also demonstrable in the rat *in vivo*. Secondly, we attempted to combine PF and CF inputs at various relative intervals to test for differential effects between these conditions. However, durable recordings of the MLIs were considerably more difficult to achieve in the rat than in the cat, so our data presentation is limited to the first 10 min after the stimulation protocols across the sample to facilitate comparisons. Figure 5 presents the average effects of the separate protocols in MLIs (in Figure 5A) and PCs (in Figure 5B). However, as the difficulties in making durable MLI recordings prevented us from obtaining a sufficient data set for each protocol type to perform satisfactory statistical tests, we also present the data in a second figure (Figure 6A) in which the effects of combined PF and CF stimulation are presented as a pooled group (regardless of the relative timing of the two stimuli).

For plasticity effects in MLIs, we obtained data from two types of stimulation protocols, one in which the CF activation coincided with the onset of the PF burst (time difference: 0 ms) and another protocol in which the CF was applied just after the termination of the PF burst (the time difference between the *onsets* of the stimulations was 150 ms). Both protocols seemed, on average, to be effective in potentiating the PF input to the MLIs with the protocol with the lagging CF activation seemingly more effective (Figure 5A). In PCs, the same protocols seemed to cause a depression of the PF inputs (Figure 5B). When the PF burst stimulation protocol was applied without CF activation, or with the CF activation preceding the onset of the PF burst with 100 ms, there was instead an apparent potentiation of the PF input.

In order to obtain a statistically testable data set for the MLIs, we pooled the two protocols combining PF and CF stimulation (yielding *N* = 6). For the PCs, only the PF protocol had a sufficiently high *N* (*N* = 6) for statistical testing. For the MLIs, a repeated measures ANOVA revealed a trend, though not significant (*p* = 0.06), suggesting that the MLI responses increased as a result of the PF + CF stimulation protocols (see Figure 6A for illustration). For the PCs, a repeated measure ANOVA, followed by *post-hoc* Tukey tests, revealed a significant potentiation effect of the PF stimulation protocol (see Figure 6B for illustration). The response differences between pre-training and all post-training time points were statistically significant (at 1 min: *p* < 0.05, 5 min; *p* < 0.05 and 10 min; *p* < 0.01).

## Discussion

The main aims of the present study were to provide data on the spontaneous firing properties of MLIs in the non-anesthetized rat *in vivo* and to repeat some of the experiments made on MLIs and PCs in the decerebrated cat using direct PF and CF stimulation. We found that in the MLIs, PF responses had a short duration and were quite powerful, whereas the CF responses were less intense but had a much longer duration. Both observations are in agreement with previous findings in the cat (Jorntell and Ekerot, 2002, 2003). The PF responses in PCs were also similar in magnitude and time course to PF responses of a previous report from the rat *in vivo* (Cerminara and Rawson, 2004). In addition, we explored some basic plasticity effects for the PF input to MLIs and PCs and found that potentiation of the input to the cells can be obtained using similar protocols previously applied in the cat *in vivo* (Jorntell and Ekerot, 2002, 2003).

A primary finding was the long lasting CF response evoked in the MLIs. In a careful analysis of putative CF responses *in vitro* (Szapiro and Barbour, 2007), the conclusion was that these responses could be due to spillover of glutamate from neighboring CF synapses made on PCs. The fact that these CF responses primarily seemed to rely on NMDA receptors (Szapiro and Barbour, 2007) could explain their apparent involvement in plasticity (Jorntell and Ekerot, 2003; Dean et al., 2010; Gao et al., 2012) as previously discussed more extensively (Jorntell et al., 2010).

Plasticity effects induced by stimulation protocols were found to be statistically significant only in the case of PCs that received PF burst stimulation alone. In these cells, PF input was significantly potentiated and thus confirmed previous observations in the cat *in vivo* (Jorntell and Ekerot, 2002). PF burst stimulation combined with CF activation seemed to result in a depression rather than a potentiation of the PF input, in agreement with previous effects in the cat and rabbit *in vivo* (Ito et al., 1982; Ekerot and Kano, 1985; Jorntell and Ekerot, 2002, 2011) and in general agreement with Safo and Regehr (2008), although the number of observations we made here was too low to allow statistical testing. With regard to MLI plasticity effects induced by paired PF and IO stimulation protocols, the data indicated that there was a trend suggesting potentiated PF input (ANOVA, *p* = 0.06). The relatively moderate and short-lasting effects in MLIs compared to the findings in the cat could be due to the fact that the significantly more difficult recording conditions in the rat forced us to limit the duration of the stimulation protocols to just one minute, rather than to use 5 and 10 min that we have done previously (Jorntell and Ekerot, 2002, 2011). Also, unlike in the cat, recording from the same MLIs for more than 15 min was in many cases not possible, thus limiting our observations to effects appearing within the first 10 min. It is possible that some of the plasticity effects would have increased over the next hour, similar to findings in the cat (Jorntell and Ekerot, 2002; Bengtsson et al., 2011).

We conclude that the basic responsive properties of the cortical neurons in the rat *in vivo* are similar to those in the cat and also that it is likely that similar mechanisms of PF input plasticity apply. Our data suggest that the temporal relationships between the PF and CF activation under which potentiation of PF input to MLIs occurs could be relatively broad, though no statistically significant plasticity effects were observed here (*p* = 0.06). Further studies are required to better understand the temporal tuning curves (Safo and Regehr, 2008) for PF-CF coincidence in the regulation of PF input plasticity in both PCs and MLIs *in vivo*.

## Methods

Twelve adult Sprague-Dawley rats weighing 300–450 g were initially sedated with an intramuscular injection of ketamine (120–160 mg/kg, Ketaminol 100 mg/ml, Intervet International, Boxmeer, Holland). Under local lidocaine anesthesia (Xylocaine, 20 mg/ml, AstraZeneca, Södertälje, Sweden), a venous cannula was inserted into the jugular vein and a deep propofol (Diprivan 10 mg/ml, AstraZeneca) anesthesia was induced and maintained by intravenous injections until the moment of decerebration. Propofol, a rapidly eliminated injection anesthetic (Cox et al., 1998), was administered continuously at 5- to 10-min intervals and usually 0.8–1 ml was administered before the decerebration. Recordings did not start until about 3 h after the decerebration, and thus the discontinuation of the propofol anesthesia, which should be enough to allow for an essentially complete elimination of the propofol (Cox et al., 1998).

The level of anesthesia before decerebration was characterized by constricted pupils and a complete muscle atonia before the administration of paralyzing agents. Cannulae were inserted into the trachea, the right jugular vein, and the right femoral vein and artery. The animals were artificially ventilated and given a continuous infusion (buffered Ringer-acetate and glucose solution). The end-expiratory CO2, blood pressure, and body temperature were continuously monitored and maintained within physiological limits (4.0–4.5%, 80–150 mmHg, and 37.0–38.5°C, respectively). All wound areas were infiltrated by lidocaine. During recordings, the animals were paralyzed with pancuronium bromide (Pavulon, Organon Teknika, Boxtel, Holland).

To access the brain stem, a small craniotomy was performed in the medial part of the left side of the skull, just rostral to the lateral suture. The dura overlying the occipital part of the occipital neocortex was cut before the blunt spatula was lowered toward the brainstem. Decerebration was carried out with a blunt spatula and a coiled metal wire that was driven through the brainstem. At the brainstem level, the transections were made just rostrally to the superior colliculus. Postmortem examination verified a complete brainstem transection. The blood pressure and the end-expiratory CO2 remained stable throughout experiments, also on noxious stimulation.

The head of the animal was fixed in a frame by ear bars covered with lignocaine and by a nose ring. To increase the mechanical stability of the brain, cerebrospinal fluid was drained through a hole in the dura between the occipital bone and the first vertebra. A craniotomy was performed to expose the left posterior lobe of the cerebellar cortex and the overlying dura was cut.

A pool of cotton-in-agar was built around the cerebrum, cerebellum, and brain stem. The pool was filled with warm paraffin oil to prevent the exposed parts from drying.

A glass-insulated tungsten microelectrode for extracellular unit recording (with an exposed tip 5–15 μm, impedance 2–6 MOhm at 1 kHz) was inserted through the surface of lobule VI. The electrode was parasagittally oriented and tilted about 30° rostrally. The electrode was advanced by a motorized micromanipulator and the depth from the surface was indicated on the electronic unit controlling the manipulator.

A coiled silver wire, coated by teflon, was put on the surface of the cerebellar cortex to stabilize the recording area. One to three homemade tungsten-in-glass microelectrodes (exposed tips 50–150 um) were inserted in the superficial cortical sheet for stimulation of PFs. Another tungsten microelectrode, rostrally oriented in the parasagittal plane was inserted through the caudal brainstem, approximately 0.5 mm from the midline, to reach the contralateral IO. All stimulation through tungsten electrodes was made with pulse widths of 0.2 ms. To have as precise activation of CFs as possible, the CF responses evoked from the microelectrode was verified to evoke low threshold CF field potentials in the recording region mapped with a surface electrode. All statistical data are reported as mean ± standard deviation.

To induce plastic changes in the PF input, we used a PF burst stimulation (100 Hz, 15 pulses) repeated at 1 Hz for 1 min, that was presented alone or in combination with single stimulation of the CFs (also repeated at 1 Hz) given at different times relative to the onset of the PF stimulation as indicated in the text. The PF burst stimulation protocol was similar to that previously used in the cat *in vivo* (Jorntell and Ekerot, 2002, 2003). Responses were taken as the net change in spike firing, i.e., we subtracted the average baseline firing of 200 ms preceding the stimulation.

In some cases stimulus artifacts (of 1–2 ms duration) overshadowed spikes and made spike identification impossible. Therefore, in the peri-stimulus time histograms, these bins were replaced with the average spontaneous activity level preceding stimulation.

All statistical values are reported as mean ± standard deviation (SD). Statistical analysis of spontaneous spike activity was performed as described in Ruigrok et al. (2011) and all values were calculated from a train of spontaneous spikes recorded for 30–60 s. The average frequency (Avg Freq) for each cell type was calculated as the population mean. For each cell, ISIs were extracted and the median ISI (ISImed) was identified. ISIs were then ordered from smallest to largest and the fifth percentile (ISIperc05) was identified. CV2 is defined as the mean of two times the absolute difference of successive ISIs, divided by the sum of both intervals. CVlog was calculated by taking the logarithm of each ISI, calculating the mean and SD values, and then dividing the SD with the mean.

In the plasticity experiments, response magnitudes to electrical sitmulations were quantified by calculating the instantaneous frequency in 0.1 ms bins and summating them over a 4 ms time window after stimulation. This time window was chosen since it represents approximately the time of integration of MLI EPSPs *in vivo*, which have a half-decay time of 4 ms (Jorntell and Ekerot, 2003).

Statistical analyses of differences in response latencies and durations were performed with Student's *t*-test (two-tailed, without assuming equal population variances). Levels of significance reported here are 0.05 (indicated by ^*^ in figures) and *p* < 0.001 (^***^).

All statistical analyses of changes in response magnitudes after stimulation protocols were performed with repeated measures ANOVAs. In the case where the ANOVA yielded a significant effect of training, a *post-hoc* Tukey's (Honestly Significant Difference) test was performed. The different levels of significance reported here are *p* < 0.05 (indicated by ^*^ in figures) and *p* < 0.01 (^**^).

The experimental procedures were approved in advance by the local Swedish Animal Research Ethics Committee.

### Conflict of interest statement

The authors declare that the research was conducted in the absence of any commercial or financial relationships that could be construed as a potential conflict of interest.
